# Vancomycin-Induced Stevens-Johnson Syndrome in a Boy Under 2 Years Old: An Early Diagnosis by Granulysin Rapid Test

**DOI:** 10.3389/fped.2018.00026

**Published:** 2018-03-13

**Authors:** You-Cheng Lin, Ji-Nan Sheu, Wen-Hung Chung, Ren-You Pan, Chu-Ju Hung, Jen-Jung Cheng, Yu-Ping Hsiao

**Affiliations:** ^1^School of Medicine, Chung Shan Medical University, Taichung, Taiwan; ^2^Department of Medical Education, Chung Shan Medical University Hospital, Taichung, Taiwan; ^3^Department of Pediatrics, Chung Shan Medical University Hospital, Taichung, Taiwan; ^4^Department of Dermatology, Drug Hypersensitivity Clinical and Research Center, Chang Gung Memorial Hospital, Linkou, Taiwan; ^5^College of Medicine, Chang Gung University, Taoyuan, Taiwan; ^6^Department of Dermatology, Chung Shan Medical University Hospital, Taichung, Taiwan

**Keywords:** vancomycin, Stevens-Johnson syndrome, granulysin rapid test, Algorithm of Drug Causality for Epidermal Necrolysis score, modified T-cell activation assay

## Abstract

Stevens-Johnson syndrome (SJS) is a life-threatening disease, which is mainly ascribed to drugs, such as sulfonamides and psychoepileptics. In this article, we present a pediatric case of vancomycin-induced SJS and an alternative diagnostic algorithm. The patient presented with multiple target-like rashes and vesicles throughout the whole body after receiving vancomycin. Despite the fact that skin biopsy remains the gold standard for diagnosing SJS, the granulysin rapid test by immunochromatographic assay is a non-invasive option for children. In this article, we describe our use of the Algorithm of Drug causality for Epidermal Necrolysis and a modified T-cell activation assay for granzyme B and interferon gamma to screen for the culprit drug. Moreover, we applied the granulysin rapid test as an early diagnosis method for children with drug-induced SJS.

## Introduction

Seldom reported in children, Stevens-Johnson syndrome (SJS) is a life-threatening disease with an incidence of 0.1–0.2 per million a year and a mortality rate of 7.5% in pediatric groups (1, 2). Cases are mainly related to drugs such as sulfonamides and psychoepileptics (2, 3). Long-term sequelae including cutaneous and ocular manifestations occur in about half of affected children, along with a high recurrence rate (20% within 7 years after the index episode). Biopsy is now the gold standard to diagnose SJS. However, it is an invasive and time-consuming procedure. On young and often resistant children, skin biopsies are difficult to perform and can also raise concern from parents. Therefore, we sought alternate methods to diagnose SJS. Herein, we describe a case of pediatric vancomycin-induced SJS and implement early diagnosis with the granulysin rapid test.

## Background

A 19-month-old boy was admitted for left-sided empyema and sepsis. Laboratory tests showed a normal leukocyte count with left shift (leukocytes 5,340/μl, band form 23%). C-reactive protein was 21.75 mg/dl. His pleural fluid was exudate according to Light’s criteria [effusion protein 4.1 g/dl, serum protein 5.1 g/dl, effusion lactate dehydrogenase (LDH) 965 IU/l, and serum LDH 269 IU/l, with the upper limit of the laboratory’s reference range of serum LDH 192 IU/l]. The patient had no underlying illness, nor did family history and personal history contribute. Empirical intravenous antibiotics with vancomycin 0.2 g every 8 h and ceftriaxone 0.5 g every 12 h were administered. The antibiotic was changed to monotherapy with vancomycin after methicillin-resistant *Staphylococcus aureus* (MRSA) was identified by pleural fluid culture. Vancomycin trough level was 16.5 µg/ml. After 13 days of vancomycin treatment, polymorphous rashes developed over his face and upper trunk. However, vancomycin was continued for a total of 20 days due to his infection status.

**Figure 1:**
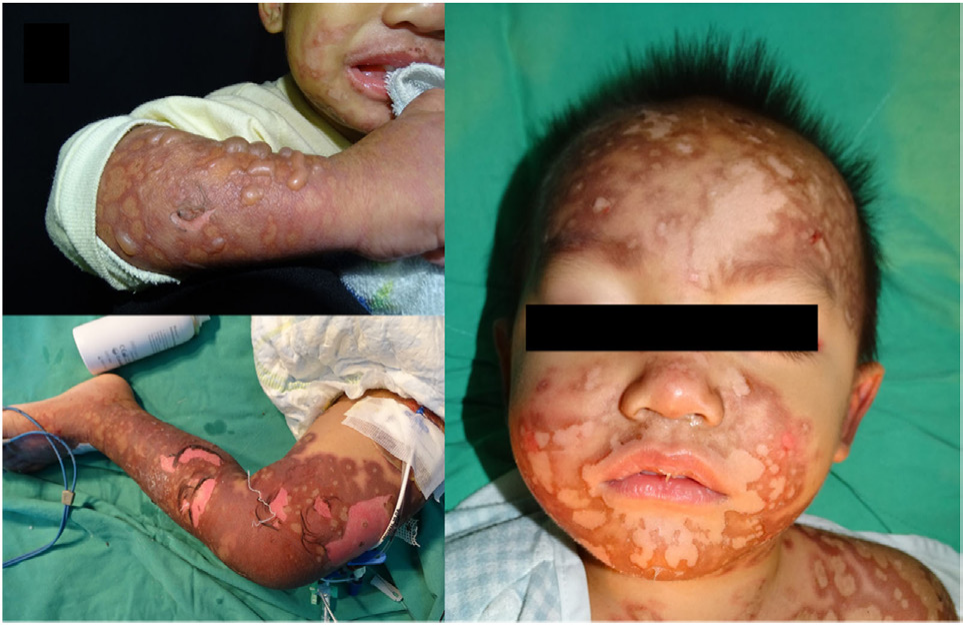
Multiple reddish macules, patches and tense bullae with atypical target lesions over the face, neck, trunk and limbs of patient. Written informed consent was obtained from the mother of the patient for publication.

**Figure 2:**
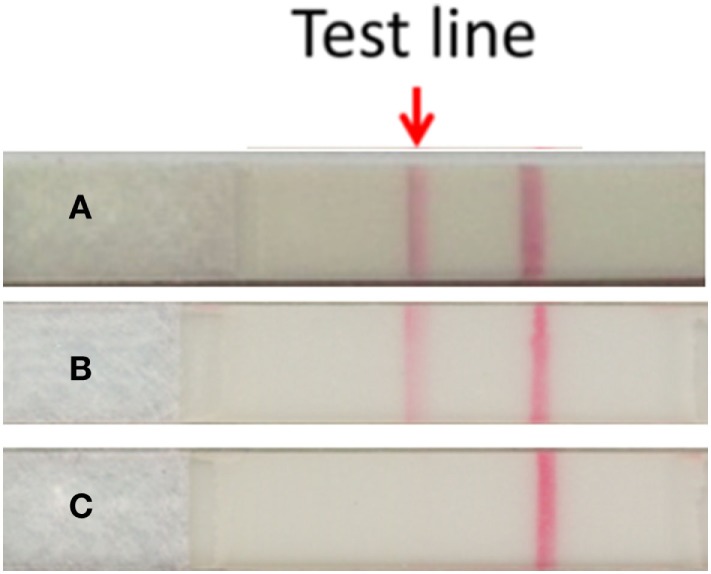
Detection of serum granulysin by the granulysin rapid test. **(A)** Serum taken from the SJS patient at the time of blister formation. **(B)** Recombinant human granulysin as positive control. **(C)** Normal human serum as negative control. The method of results interpretation is according to Fujita et al. (4).

On day 3 after finishing vancomycin treatment, multiple and progressive target-like macules and papules developed across the patient’s lower trunk and limbs, and then extended to his forehead, lips, whole trunk, and extremities (Figure 1). Tense blisters emerged on the erythematous background lesions over his extremities and involved 10% of the total body surface area. Erosions of mucous membranes were found in his oral cavity, conjunctivae, and genital area. When target lesions developed, we used the granulysin rapid test, an immunochromatographic assay described by Fujita et al. (4), to diagnose SJS by serum. Briefly, this granulysin rapid test utilizes two monoclonal antibodies specific to granulysin, RB1 and RC8 (from MBL, Nagoya, Japan). Granulysin in the serum sample binds to RB1 and conjugates with microparticles, while RC8 is immobilized to form a result line. Granulysin and RB1 comigrate upward *via* microparticles until the granulysin is sandwiched with RC8. Approximately 10 ng/ml of sample yields a result line within 15 min. In our case, the granulysin level in serum revealed a positive result consistent with an attack of SJS (Figure 2). Following the Algorithm of Drug causality for Epidermal Necrolysis (ALDEN), we calculated a score of 3 corresponding to “possible culprit drug” for vancomycin. We then used the modified T-cell activation assay with the enzyme-linked immunospot (ELISpot) assay for both granzyme B and interferon gamma (IFN-γ) to confirm the culprit drug. The modified T-cell activation assay was done on T cells pretreated with the suspected drugs, vancomycin and ceftriaxone. The results identified vancomycin as the culprit drug.

**Figure 3:**
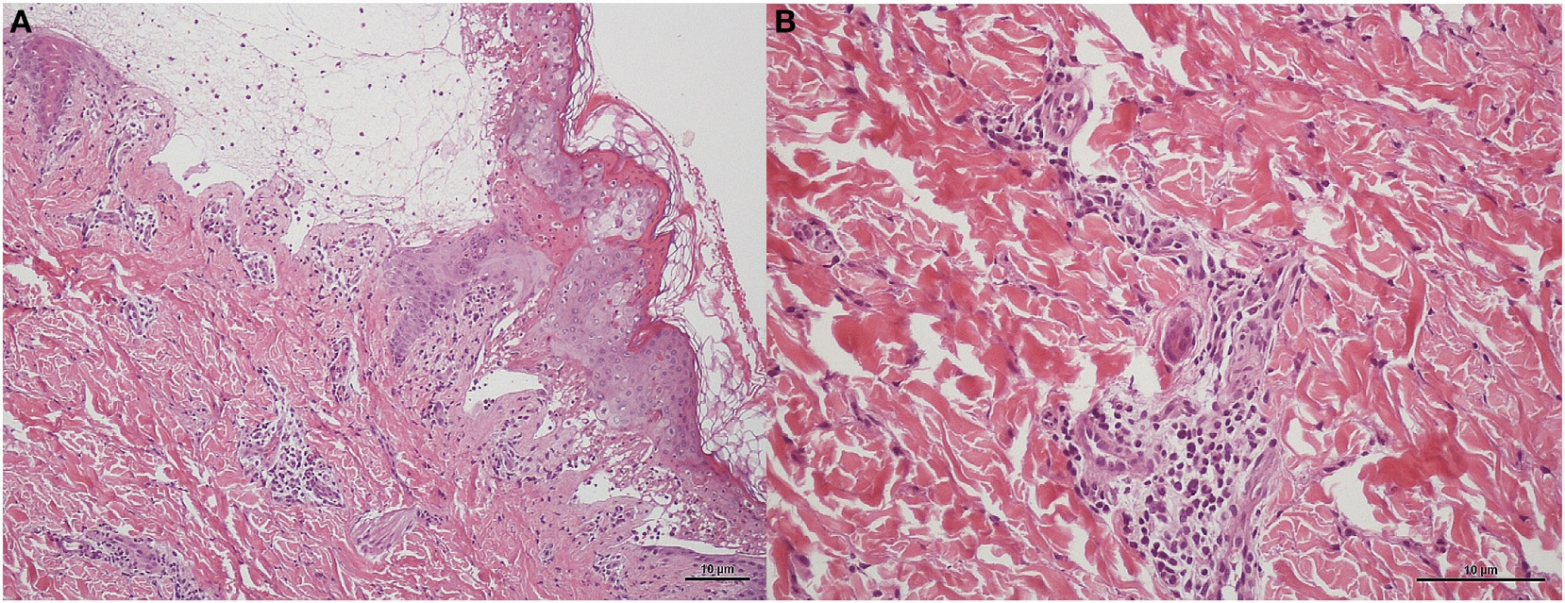
Skin biopsy of patient in H&E staining. **(A)** Subepidermal vesicles were accompanied by apoptotic keratinocytes presenting individually and in clusters within the epidermis in 10 power view. Lymphocytic infiltrations were observed scattered in the basal layer of the epidermis and around the vessels within the dermis. **(B)** Perivascular infiltrate composed primarily of lymphocytes in 20 power view.

Immediately after the rapid testing, we gave intravenous hydrocortisone 5 mg/kg/dose per 6 h with tapering. The patients’s skin biopsy later revealed typical pathologic findings of SJS, including subepidermal vesicles containing apoptotic keratinocytes and perivascular lymphocytic infiltration (Figure 3). The rashes gave way to skin desquamation, at which point the patient was discharged (Figure S1 in Supplementary Material).

## Discussion

Vancomycin is widely used against bacteria resistant to penicillin antibiotics such as MRSA. Common adverse reactions are fever, rashes, and anaphylaxis (5). Other adverse reactions include the red man syndrome, thrombophlebitis, ototoxicity, and nephrotoxicity. Dermatological side effects of vancomycin include SJS, exfoliative dermatitis, Drug Rash with Eosinophilia and Systemic Symptoms (DRESS) syndrome, linear IgA bullous dermatitis, and leukocytoclastic vasculitis (5).

Reports of vancomycin-induced SJS in the literature are limited, and in Table 1 we show four cases (6–9). Three patients were adults, while one case was a 3-year-old child. The polymorphous rashes over face and upper trunk in our patient are considered an atypical pattern of SJS and have a 10-day latency before target lesions developed. The 10-day latency may be attributed to the vancomycin dosage. Considering the patient’s body weight (10 kg), our patient received a relatively high dosage of vancomycin (0.6 g daily) compared to the 1.5 and 2 g daily reported for adult patients (6, 9). Among the mentioned reports, a 74-year-old patient shared a 10-day latency with our patient (9), while the 3-year old child developed new blisters over his lower extremities on day 10 of hospitalization, 5 days after his last IVIG infusion (8). As these patients share an immune-compromised background, vancomycin’s cytotoxic effects are more pronounced and cause the rashes. The atypical lesions and latency can lead to misdiagnosis before typical characteristics of SJS appear, for instance the target-like lesions appearing on day 23 in this case. Such situations may delay the suspension of vancomycin.

**Table 1:** Reported cases of vancomycin-implicated SJS.

Reference	Age, sex	Race	Underlying disease	Other drugs	Rashes (days)^a^	Vesicles (days)^b^
Alexander II et al. (6)	36 years, M	Caucasian	Endocarditis	No	17	27

Laurencin et al. (7)	71 years, M	Not stated	1. Rheumatoid arthritis 2. Cervical fusion wound infection	Ciprofloxacin	29	29

Metry et al. (8)	3 years, M	Black	Not stated	Not stated	Not stated	Not stated

Yang et al. (9)	74 years, M	Han Chinese	1. COPD 2. Rectal cancer 3. Endocarditis	1. Teicoplanin 2. Moxifloxacin 3. Biapenem	2 days after Moxifloxacin (14 days)	7

Our patient	2 years, M	Han Chinese	Pneumonia	Ceftriaxone	13	23

*^a^This is the period from initiation of a first course of vancomycin to onset of rash*.

*^b^This is the period from initiation of a first course of vancomycin to onset of vesicles*.

*M, male; F, female; COPD, chronic obstructive pulmonary disease*.

Biomarkers may be a feasible solution for early detection of SJS before performing skin biopsy. Notably, granulysin has been shown to be an important mediator contributing to keratinocyte death in SJS (10). Abe et al. (11) reported that the level of serum granulysin in patients with early-stage SJS was higher than those in other drug-induced skin reactions. Fujita et al. (4) pioneered the use of the granulysin rapid test by immunochromatographic assay for early diagnosis of SJS and the closely associated toxic epidermal necrolysis (TEN). We used the same kit described by Fujita et al. (4), which has a sensitivity of 80% and a specificity of 95.8% for SJS/TEN (*P* = 1.02 × 10^−3^, analyzed by Fisher’s exact probability test). In our case, we performed the granulysin rapid test when the patient developed target lesions. The preliminary positive diagnosis was later confirmed by skin biopsy. Indeed, despite skin biopsy being the current gold standard to diagnose SJS, it is invasive, time-consuming and of concern to parents when young children are involved. Therefore, we suggest the use of the granulysin rapid test as a non-aggressive alternative option adjunct to skin biopsy, with the additional advantage of rapid diagnosis within 15 min.

To screen suspected culprit medications, ALDEN scores were calculated (12). Five items were considered in the algorithm: index day, half-life, prechallenge/rechallenge, dechallenge, and notoriety. Following the algorithm, all the prescribed drugs of our patient were assessed and vancomycin was highly suspected (ALDEN score 3 points for vancomycin and only 1 for ceftriaxone).

The T-cell activation assay was recently suggested as an alternative for the lymphocyte transformation test to screen for culprit drugs, with a sensitivity of 80% (95%CI: 52–96%) and specificity of 96% (95%CI: 80–99%) (13). The assay involves three different protocols to examine the expression of the cytokines: granulysin expression in CD4^+^ cells by flow cytometry, granzyme B production by ELISpot assay, and IFN-γ levels in cell supernatant by cytokine bead array. We performed a modified T-cell activation assay which involved the ELISpot assay of both granzyme B and IFN-γ on T cells pretreated with the suspected drugs. This provided us with the expression profiles of the two cytokines simultaneously, which saved both time and cost compared with the original protocols. Our patient showed higher expression of both granzyme B and IFN-γ in T cells pretreated with vancomycin, while only granzyme B was expressed in T cells pretreated with ceftriaxone. These results pointed to vancomycin as the causative drug for our patient.

Table 1 shows select reported cases of vancomycin-induced SJS. According to the study by Levi et al. (2), the incidence of SJS increases with age and is seldom seen in children less than 15 years old. While impurities in early vancomycin production once contributed significantly to adverse reactions, a history of multiple exposures is now considered a more relevant factor (5). Since it was our patient’s first exposure to vancomycin, we surmise that he may carry other risk factors for SJS. In Han Chinese, for example, strong associations between HLA alleles and SJS have been reported (14, 15). The possibility that our Han Chinese patient may carry genetic variations with similar mechanisms calls for further investigation.

## Concluding Remarks

In this article, we report a case of vancomycin-induced SJS in a child and the use of the granulysin rapid test as an attractive alternative to traditional skin biopsy for SJS diagnosis, with rapid result time being a major advantage. We also describe the combined use of the ALDEN score and the modified T-cell activation assay to efficiently identify the culprit drug. These methods open a route for early diagnosis without skin biopsy for drug-induced SJS, accompanied by appropriate countermeasures against a prolonged course. While more research is needed to build a novel diagnosis process, we are hopeful that the methods presented here will contribute to better understanding, diagnosis, and management of drug-induced SJS.

## Ethics Statement

This study was carried out in accordance with the policy of the Ethics Committee, Chung Shan Medical University Hospital, Taiwan, with written informed consent from the mother of the patient. The mother of the patient gave written informed consent in accordance with the Declaration of Helsinki. Consent was obtained from the mother for the incompetent pediatric patient.

## Author Contributions

Y-CL conceptualized and drafted the initial manuscript. J-NS and R-YP critically reviewed and revised the manuscript. W-HC carried out the modified T-cell activation assay to make the diagnosis and critically revised the manuscript. C-JH and J-JC helped with the therapy and follow-up of the patient, and with writing of the initial manuscript. Y-PH was involved in making the diagnosis, follow-up of the patient, and critically revising the manuscript. All authors approved the final manuscript as submitted and agree to be accountable for all aspects of the work.

## Conflict of Interest Statement

The authors declare that the research was conducted in the absence of any commercial or financial relationships that could be construed as a potential conflict of interest.

## Supplementary Material

Figure S1 Skin pattern before treatment **(A)** and after treatment **(B)**.

## Abbreviations

SJS, Stevens-Johnson syndrome; LDH, lactate dehydrogenase; MRSA, methicillin-resistant *Staphylococcus aureus*; ALDEN, Algorithm of Drug Causality for Epidermal Necrolysis; TEN, toxic epidermal necrolysis; IFN-γ, interferon gamma.
