# Supplementary figures and images for: Vancomycin-Induced Stevens-Johnson Syndrome in a Boy Under 2 Years Old: An Early Diagnosis by Granulysin Rapid Test
Source: Front Pediatr. 2018 Mar 13;6:26. doi: 10.3389/fped.2018.00026 (PMC5859217; doi:10.3389/fped.2018.00026)

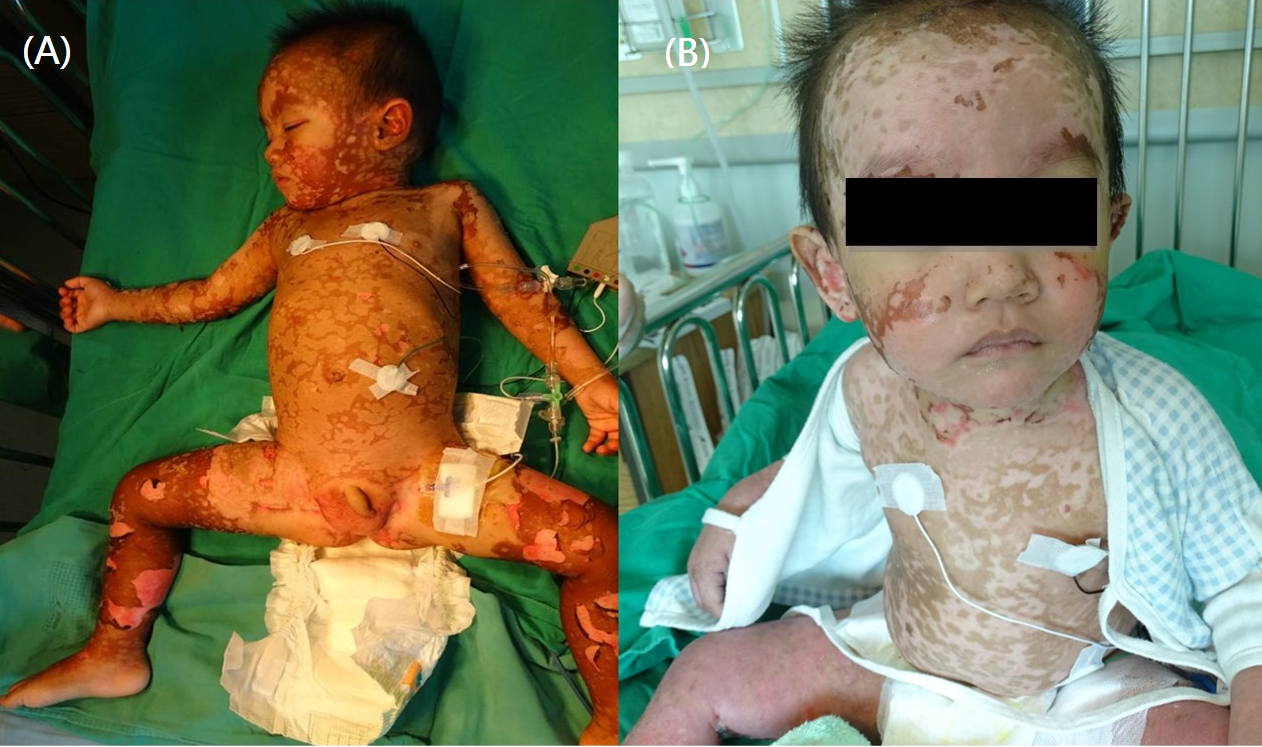

Supplement: Figure S1 — Skin pattern before treatment (A) and after treatment (B). [file image_1.TIF]
